# The profile and contribution of rare germline copy number variants to cancer risk in Li-Fraumeni patients negative for *TP53* mutations

**DOI:** 10.1186/1750-1172-9-63

**Published:** 2014-04-28

**Authors:** Amanda G Silva, Ana CV Krepischi, Peter L Pearson, Pierre Hainaut, Carla Rosenberg, Maria Isabel Achatz

**Affiliations:** 1 Department of Genetics and Evolutionary Biology, Institute of Biosciences, University of São Paulo, Rua do Matão, 277-05422-970 São Paulo, São Paulo, Brazil; 2 International Center for Research and Training, A. C. Camargo Cancer Center, Rua Taguá, 440-01508-010 São Paulo, Brazil; 3 International Prevention Research Institute, 15 Chemin du Saquin, 69130 Ecully, France; 4 Department of Oncogenetics, International Center for Research and Training, A. C. Camargo Cancer Center, Rua Taguá, 440-01508-010 São Paulo, Brazil

**Keywords:** Li Fraumeni syndrome, Rare CNVs, Familial cancer

## Abstract

**Background:**

The Li-Fraumeni syndrome (LFS) is an inherited rare cancer predisposition syndrome characterized by a variety of early-onset tumors. Although germline mutations in the tumor suppressor gene *TP53* account for over 50% of the families matching LFS criteria, the lack of *TP53* mutation in a significant proportion of LFS families, suggests that other types of inherited alterations must contribute to their cancer susceptibility. Recently, increases in copy number variation (CNV) have been reported in LFS individuals, and are also postulated to contribute to LFS phenotypic variability.

**Methods:**

Seventy probands from families fulfilling clinical criteria for either Li-Fraumeni or Li-Fraumeni-like (LFS/LFL) syndromes and negative for *TP53* mutations were screened for germline CNVs.

**Results:**

We found a significantly increased number of rare CNVs, which were smaller in size and presented higher gene density compared to the control group. These data were similar to the findings we reported previously on a cohort of patients with germline *TP53* mutations, showing that LFS/LFL patients, regardless of their *TP53* status, also share similar CNV profiles.

**Conclusion:**

These results, in conjunction with our previous analyses, suggest that both *TP53*-negative and positive LFS/LFL patients present a broad spectrum of germline genetic alterations affecting multiple loci, and that the genetic basis of LFS/LFL predisposition or penetrance in many cases might reside in germline transmission of CNVs.

## Introduction

Li-Fraumeni syndrome (LFS) is an inherited condition characterized by early-onset sarcoma, brain, breast and other cancers. Families with incomplete LFS features are referred to as Li-Fraumeni-like (LFL) [1,2]. Germline mutations in the tumor suppressor gene *TP53* account for over 50% of the families matching LFS criteria [3] but for only 20-40% of the LFL families [4]; lack of *TP53* mutation in a significant proportion of LFS/LFL families, suggests that other types of inherited alterations must contribute to their cancer susceptibility. Although point mutations have been commonly described, DNA copy number variations (CNVs) have been reported as an alternative mechanism for cancer predisposition for at least 30% of known Mendelian cancer genes [5,6], including *TP53*[7,8], *APC*[9], *BRCA1*[10] and the mismatch repair gene *MSH2*[9].

We used microarray-based comparative genomic hybridization (array-CGH) to screen for CNVs in the germline DNA of 70 patients fulfilling diagnostic criteria for LFS or LFL, but with no detectable mutation involving *TP53*[11]. Results were compared to a random sample of 100 Brazilian control individuals [12], to a sample of LFS/LFL *TP53* mutated patients previously published by us [13], and to publically available CNV data in normal individuals (DGV).

## Subjects and methods

### Patients

The patients were recruited and ascertained at the Department of Oncogenetics of the A. C. Camargo Cancer Center, São Paulo, Brazil. The protocol was approved by the ethics committee of the institution and informed consent obtained from all subjects and their families. DNA was isolated from peripheral leukocytes using standard protocols. The cohort comprised 70 non-related probands fulfilling either the classical definition of LFS or at least one of the clinical criteria commonly defining LFL (Chompret, Birch or Eeles’s definitions) [14-17]. These DNA samples had been previously shown to have no mutations in the coding sequences (exons 2 to 11) or splice junctions of the *TP53* gene [18].

### Controls

The CNV data of a group of 100 individuals randomly selected from the urban area of São Paulo, Brazil, was used as control for this study as previously described [12].

#### Array-CGH

Array-CGH was performed using a 180 K whole-genome platform (design 22060, Agilent Technologies, Santa Clara, USA), with an average spacing of 18 Kb between probes. Scanned images of the arrays were processed and analyzed using Feature Extraction software and Genomic Workbench software (both from Agilent Technologies), together with the statistical algorithm ADM-2, and using a sensitivity threshold of 6.7. We applied a ‘loop design’ in our hybridizations as previously described [19], resulting in two reverse labeling hybridizations per sample. Alterations had to encompass at least three consecutive probes with aberrant log_2_ values to be called by the software, and those not detected in both dye-swap experiments were excluded from the analysis.

#### Analysis

The detected copy number variations were compared to CNVs reported in the Database of Genomic Variants (DGV; *http://dgv.tcag.ca/dgv/app/home*; freeze December, 2011). We arbitrarily classified CNVs into “rare” and “common” by considering as “rare” those CNVs encompassing coding sequences and present in frequencies < 0.1% in DGV, that does not exclude that some rare CNVs might contain smaller segments which can vary in copy number in the population. Mann–Whitney and Fisher-exact tests were used to compare patients and controls for frequency, gene density and size distribution of CNVs.

Gene annotation was performed using the University of California Santa Cruz Genome Browser (UCSC) and the Catalog of Somatic Mutations (COSMIC; http://cancer.sanger.ac.uk/cancergenome/projects/cosmic/).

## Results

The clinical classification of the patients, description of their cancer types and age at onset are given in Additional file 1: Table S1. A total of 567 CNVs were identified in the 70 patients investigated (308 losses and 259 gains). Table 1 presents the CNV features detected in the LFS/LFL patients without *TP53* mutations, the previously published CNV data from LFS/LFL patients with mutation [13] and controls. Full CNV data are presented in Additional file 2: Table S2.

**Table 1:** **Features of the detected CNVs in *TP53 *** **mutated and non-mutated patients compared to controls: size, number of genes, and gene density**

	***TP53 *** **type of mutation**	**Number of individuals**	**Number of CNVs (rare/common)**	**Mean number of common CNVs**	**Mean size of common CNVs (Kb)**	**Gene density of common CNVs (gene number/Mb)**	**Mean number of rare CNVs**	**Mean size of rare CNVs (Kb)**	**Gene density of rare CNVs (gene number/Mb)**
***TP53- *** mutated*	*DNA binding domain*	9	10/56	7.33 ± 2.5	165.0 ± 233.3	14.0	1.11 ± 1.2 (p = 0.0086)	168.7 ± 202.8	15.4 (p = 0.0159)
	*R337H*	12	3/89	7.67 ± 2.1	186.6 ± 343.0 (p = 0.0032)	10.4	0.25 ± 0.6	204 ± 161.6	8.1
	*All*	21	13/145	7.6 ± 2.3	178.2 ± 304.6 (p = 0.0022)	12.8	0.65 ± 1.0	176.8 ± 188.3	13.4 (p = 0.0367)
***TP53***-**wild type**		70	40/527	7.5 ± 2.6	208.7 ± 459.9 (p = 0.0008)	9.0	0.57 ± 0.9 (p = 0.0145)	94.5 ± 101.8 (p = 0.0001)	31.4 (p = 0.0001)
**Controls**		100	23/679	6.7 ± 3.0	236.7 ± 454.0	8.8	0.23 ± 0.4	232.2 ± 152.7	8.6

*Previously published results [13].

**Figure 1:**
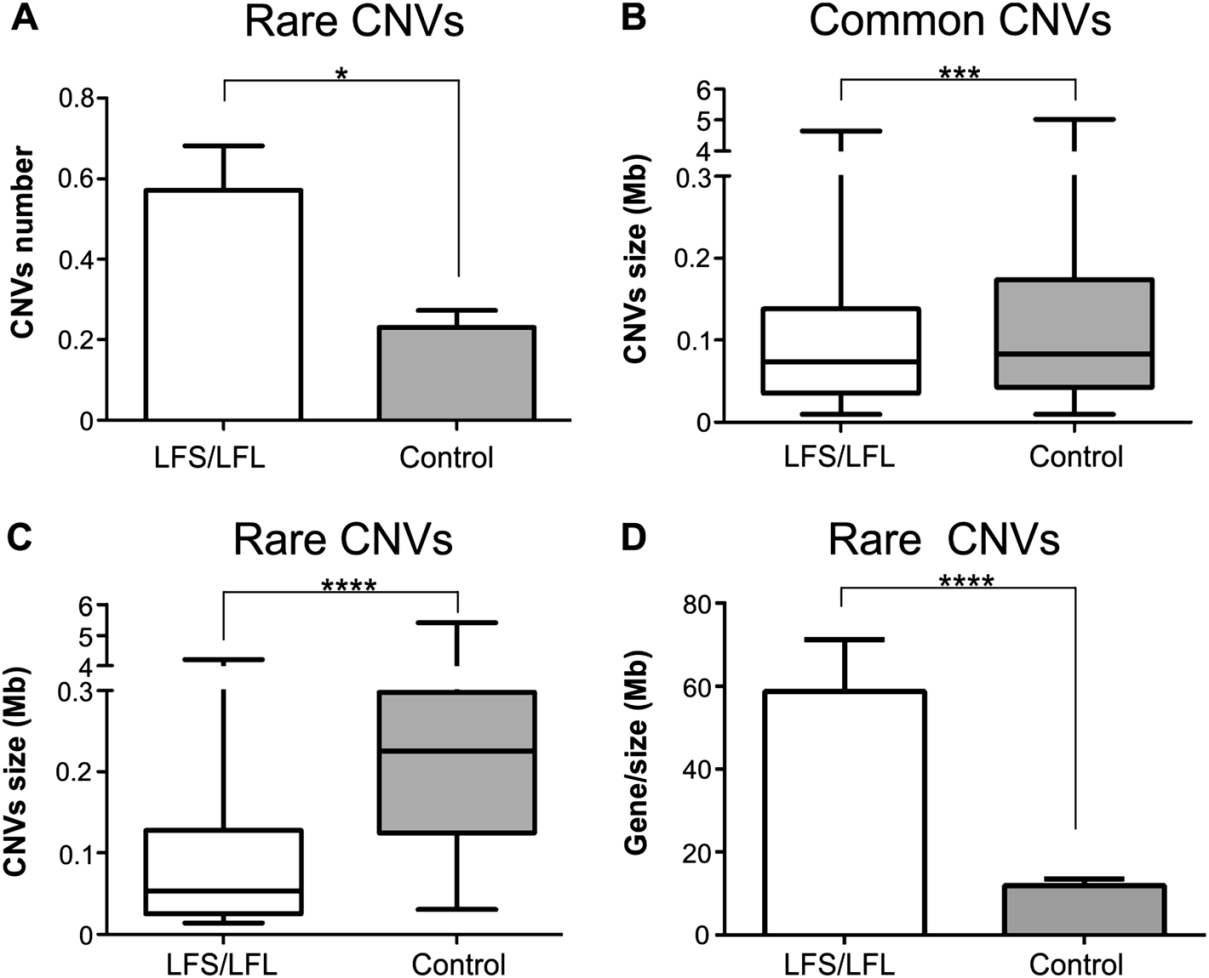
**Comparison between the CNVs in Li-Fraumeni patients and controls. (A)** Frequency of rare CNVs; **(B)** Size of common CNVs; **(C)** Size of rare CNVs; **(D)** Gene density in LFS patients and controls. Mann-Whitney test; *p =0.014; ***p < 0.001, ****p < 0.0001.

No significant differences were found between the *TP53-*negative patients compared to controls regarding the number of common CNVs per genome or ratio of losses to gains. However, there was a significantly higher number of rare CNVs per genome in this patient group (p = 0.014; Mann–Whitney test) (Figure 1A). With respect to CNV size, the CNVs in the *TP53-*negative patients were significantly smaller than controls both for common (Figure 1B) and rare (Figure 1C) CNVs (p < 0.001; Mann–Whitney test).

Furthermore, the mean number of genes encompassed by rare alterations per Mb (gene density) was much higher in patients with 31 genes per Mb as opposed to 9 in controls (p < 0.0001; Mann Whitney test) (Figure 1D).

In addition, while no recurrence was found among the rare CNVs in control individuals, 4 rare CNV regions were either recurrent or partially overlapping in 2 independent patients for each region (Table 2).

**Table 2:** **Recurrent CNV regions in patients without *TP53 *** **mutation**

**Chr**	**Start position**	**End position**	**Type**	**Size**	**Genes associated**	**Patient**	**Tumor**	**Age of onset**	**Tumor**	**Age of onset**
**chrX**	148653235	148789920	Gain	136685	HSFX1, HSFX1, MAGEA9, MAGEA8	Y0123T000	Breast	37	Breast	37
**chrX**	148638057	148835731	Gain	197674	HSFX1, HSFX1, MAGEA9, MAGEA8	Y006T00	STS	15	Breast	32
**chr19**	2173067	2187671	Gain	14604	DOT1L, PLEKHJ1, hsa-mir-1227	Y101T000	Breast	48	Thyroid	52
**chr19**	2173067	2187671	Loss	14604	DOT1L, PLEKHJ1, hsa-mir-1227	Y0114T000	Osteosarcoma	30		
**chr5**	140207385	140243110	Gain	35725	PCDHA11, PCDHA12, PCDHA13	Y0110T000	Breast	36		
**chr5**	140207385	140243110	Loss	35725	PCDHA11, PCDHA12, PCDHA13	Y0112T000	Breast	34		
**chr7**	2182941	2234914	Gain	51973	MAD1L1	Y0143T000	Breast	42		
**chr7**	2182941	2244756	Loss	61815	MAD1L1, FTSJ2	Y0123T000	Bilateral breast	37, 37		

STS – soft tissue sarcoma.

## Discussion

Despite extensive search for other genes underlying LFS/LFL, no genes other than *TP53* have been consistently associated with this complex syndrome. However, patient series in the US and Europe have shown that only ~ 30% of those subjects tested for *TP53* mutation because of familial predisposition or early-onset of cancer turned out to be positive. This strongly suggests that genetic factors other than *TP53* mutations must be contributing to familial predisposition cancer in many LFS/LFL subjects.

We previously described an increased number of CNVs in Brazilian Li-Fraumeni patients carrying germline mutations in the *TP53* gene, and also reported an increased number of rare CNVs per genome in patients carrying mutations that affected the DNA binding domain (DBD) of the *TP53* gene compared to both controls and p.R337H mutants [13]. The Brazilian founder mutation p.R337H has a markedly less severe impact on tumor predisposition [20,21] and has a CNV profile much closer to controls than the *TP53* DBD mutations [13]. A similar, but milder increase in number of rare CNVs was observed among non-mutated patients. In a previous article [13], we speculated that an increase of rare CNVs could result from inefficient selection against pathogenic CNVs, due to failure or reduction in apoptosis driven by *TP53* germline mutations. Similarly, in this new study of *TP53-*negative patients, increases in the number of rare CNVs could result from mutations in genes other than *TP53*, possibly in the *TP53* pathway, as previously suggested [22-24]. Such an example was recently reported by us [25] in a patient of the present cohort and involved the deletion of the full *BAX* gene, which is directly activated by the TP53 protein. The very high gene content of rare CNVs in *TP53*-negative patients (an almost four-fold increase compared to controls) suggests that these CNVs are under strong selective pressure, and that their gene content likely contribute to oncogenesis. Interestingly, *TP53*-mutated patients also exhibit increased gene density, although this phenomenon is much less striking than in the non-mutated patients (Table 1). These results show that LFS/LFL patient’s, with the exception of those carrying the p.R337H mutation, share similar CNV profiles regardless of their mutation status.

The recurrent regions detected in the rare CNVs in our study encompass several genes with potential functional relevance to carcinogenesis, including *MAD1L1, DOT1L1, MAGEA8* and *MAGEA9. MAD1L1* encodes a protein that plays an important role in maintaining spindle checkpoint functions and alterations in this gene have been associated with colon, lung, prostate and breast cancers [26,27]. The histone methyltransferase *DOT1L1* is involved in leukemia [28]. The *MAGE* gene family encodes proteins only expressed on normal germ cells of the testis but are ectopically expressed in melanomas and in a variety of other common cancer types [29]. Although the frequency of each recurrent region is individually low, as a group, rare CNVs may represent a significant contributor to the etiology of LFS/LFL.

Although the potential role of CNVs as genetic risk factors to cancer predisposition has not yet been fully defined, there is now compelling evidence that cancer-related genes may be encompassed or overlapped by common CNVs. Shlien et al. [30] found a significant enrichment of CNVs in LFS probands; among the genes encompassed by common CNVs, they reported recurrence of a duplication of *MLLT4*, a target of the RAS pathway. In agreement with this, we also detected common *MLLT4* duplications in three of the *TP53-*negative patients but none in the controls. We have also recently reported a 691 kb recurrent deletion at 7q34 harboring only the *PIP* and *TAS2R39* genes [31] in five patients with high cancer predisposition from different cohorts, including two *TP53*-negative patients from the present study and one *TP53*-positive LFS patient. Common cancer CNVs, such as the ones harboring *MLLT4* and *PIP*, most likely confer a minor increase in disease risk that collectively or in association with highly penetrant mutations may cause a substantially elevated risk.

## Conclusion

These findings support the hypothesis that in LFS/LFL families not carrying *TP53* mutations, cancer predisposition may be caused by a broad spectrum of genetic alterations affecting multiple loci. How CNVs and other genetic modifiers interact and modulate *TP53* tumor suppressor activities remain to be determined. Elucidating these mechanisms may hold the key to define evidence-based strategies for counseling using combined risks from *TP53* and other variants, including CNVs.

## Availability of supporting data

The data set supporting the results of this article is included within the article and its additional files.

## Abbreviations

LFS: Li-Fraumeni syndrome; LFL: Li-Fraumeni like syndrome; CNV: Copy number variation.

## Competing interests

The authors declare that they have no competing interests.

## Authors’ contributions

AGS carried out the molecular genetic studies. AGS and PLP wrote the manuscript. ACVK and CR participated in the design and coordination of the study and helped writing the manuscript. MIWA recruited and selected the patients. All authors read and approved the final manuscript.

## Supplementary Material

Additional file 1: Table S1 Clinical classification, gender, cancer type and age at onset of the patients. Click here for file

Additional file 2: Table S2 Full CNV data on the 70 probands, rare CNVs in bold-chromosome coordinates given according to Hg18. Click here for file
