# Supplementary material for: The profile and contribution of rare germline copy number variants to cancer risk in Li-Fraumeni patients negative for TP53 mutations
Source: Orphanet J Rare Dis. 2014 Apr 28;9:63. doi: 10.1186/1750-1172-9-63 (PMC4022048; doi:10.1186/1750-1172-9-63)
Supplement: Additional file 1: Table S1 — Clinical classification, gender, cancer type and age at onset of the patients. [file 1750-1172-9-63-S1.doc]

Supplementary Material 1 - Clinical classification, gender, cancer type and age at onset of the patients.

| **ID** | **Classification** | **Gender** | **tumor type (age of onset)** |
| --- | --- | --- | --- |
| **Y2T0** | Eeles2 | F | STS (42) |
| **Y3T0** | Eeles1 | F | Breast (42) |
| **Y5T0** | Eeles1 | F | CRC (60) |
| **Y6T0** | Eeles2 | F | Breast (32) |
| **Y9T0** | LFS | F | STS (18); STS (25); Breast (46) |
| **Y10T0** | Eeles1 | F | STS (55) |
| **Y11T0** | Eeles1 | F | Thyroid (73); Melanoma (75) |
| **Y14T0** | Chompret | M | STS (36); Lymphoma (55); Prostate (63) |
| **Y16T0** | Chompret | F | CRC (55); STS (72) |
| **Y17T0** | Eeles1 | F | Breast (43, 46) |
| **Y21T0** | Chompret | M | STS (40, 44, 44, 45) |
| **Y22T0** | Chompret | M | STS (11) |
| **Y23T0** | Chompret | M | Astrocytoma (1) |
| **Y24T0** | Birch | F | Breast (79); Lymphoma (73) |
| **Y29T0** | LFS | F | Osteosarcoma (19); STS (23); Head/Neck (24); Breast (26) |
| **Y30T0** | Birch | F | Lymphoma (25) |
| **Y31T0** | Birch | M | Brain (34) |
| **Y32T0** | Birch | F | STS (56) |
| **Y34T0** | Birch | F | STS (18) |
| **Y36T0** | Birch | F | Breast (44) |
| **Y38T2** | Eeles1 | F | Melanoma (35) |
| **Y41T0** | Chompret | F | Osteosarcoma (8); Breast (28) |
| **Y42T0** | Eeles2 | M | Melanoma (51); STS (52) |
| **Y43T0** | Eeles2 | M | STS (22) |
| **Y46T0** | Eeles1 | M | STS (25); Skin (35) |
| **Y51T0** | Eeles1 | F | Breast (53) |
| **Y56T0** | Birch | F | Lymphoma (24); Osteosarcoma (24) |
| **Y63T0** | Eeles2 | F | Melanoma (30) |
| **Y82T0** | Chompret | M | Adrenal (1) |
| **Y83T0** | Chompret | F | STS (21); Breast (45) |
| **Y85T0** | Chompret | F | CRC (46) |
| **Y88T0** | Eeles2 | M | Leukemia (32) |
| **Y93T0** | LFS | F | Breast (42) |
| **Y95T0** | Eeles2 | F | Breast (36) |
| **Y101T0** | Eeles1 | F | Breast (48); Thyroid (52) |
| **Y104T0** | Birch | F | Lymphoma (31); Lung (47) |
| **Y105T0** | LFS | F | CRC (27) |
| **Y108T0** | Eeles2 | F | STS (41); Renal (43) |
| **Y110T0** | Eeles1 | F | Breast (36) |
| **Y111T0** | Eeles1 | F | STS (45) |
| **Y112T0** | Chompret | F | Osteosarcoma (30) |
| **Y114T0** | Chompret | F | Breast (48) |
| **Y115T0** | Chompret | F | Breast (36) |
| **Y116T0** | Eeles2 | F | Breast (48) |
| **Y117T0** | Eeles1 | F | Breast (44) |
| **Y118T0** | Chompret | F | STS (40); Breast (53) |
| **Y120T0** | Chompret | F | Renal (44); Thyroid (45); Leukemia (54) |
| **Y121T0** | Chompret | F | Lymphoma (54); Gynecologic (70) |
| **Y122T0** | Eeles1 | F | Breast (61); CRC (68) |
| **Y123T0** | Eeles1 | F | Bilateral Breast (37) |
| **Y124T2** | Eeles1 | M | Astrocytoma (7) |
| **Y125T0** | Eeles1 | F | Melanoma (50); CRC (57) |
| **Y126T0** | Chompret | F | Lymphoma (23); Breast (39) |
| **Y129T0** | Eeles1 | F | Thyroid (43) |
| **Y133T1** | Eeles1 | M | Bladder (21) |
| **Y134T0** | Eeles1 | F | Thyroid (46) |
| **Y135T0** | Eeles1 | F | Bilateral Breast (30) |
| **Y136T0** | LFS | F | Lymphoma (36) |
| **Y137T0** | LFS | M | Renal (3); Embrionary (4) |
| **Y138T0** | Eeles1 | M | Prostate (64) |
| **Y139T1** | Birch | F | Breast (36) |
| **Y140T0** | Chompret | M | STS (48) |
| **Y142T0** | chompret | F | Pancreas (52) |
| **Y143T0** | Eeles 2 | F | Breast (62) |
| **Y145T0** | Chompret | F | Breast (36) |
| **Y147T0** | Chompret | F | Breast (35), Melanoma (36) |
| **Y148T0** | Birch | F | Breast (32) |
| **Y150T0** | Chompret | F | STS (26) |
| **Y151T0** | Chompret | F | STS (65) |
| **Y152T0** | LFS | F | Skin (36); Breast (38) |
